# Tandem-Repeat Patterns and Mutation Rates in Microsatellites of the Nematode Model Organism *Pristionchus pacificus*

**DOI:** 10.1534/g3.112.003129

**Published:** 2012-09-01

**Authors:** Ruxandra I. Molnar, Hanh Witte, Iris Dinkelacker, Laure Villate, Ralf J. Sommer

**Affiliations:** Max-Planck Institute for Developmental Biology, Department for Evolutionary Biology, Spemannstrasse 37, D-72076 Tübingen, Germany

**Keywords:** mutation accumulation lines, *Pristionchus pacificus*, microsatellite markers, tandem repeats, *Caenorhabditis elegans*

## Abstract

Modern evolutionary biology requires integrative approaches that combine life history, population structure, ecology, and development. The nematode *Pristionchus pacificus* has been established as a model system in which these aspects can be studied in one organism. *P. pacificus* has well-developed genetic, genomic, and transgenic tools and its ecologic association with scarab beetles is well described. A recent study provided first mutation rate estimates based on mitochondrial genome sequencing and mutation accumulation line experiments that help resolve rather ancient evolutionary branches. Here, we analyzed the tandem-repeat pattern and studied spontaneous mutation rates for microsatellite markers by using the previously generated mutation accumulation lines. We found that 0.59%–3.83% of the genome is composed of short tandem repeats. We developed 41 microsatellite markers, randomly chosen throughout the genome and analyzed them in 82 mutation accumulation lines after 142 generations. A total of 31 mutations were identified in these lines. There was a strong correlation between allele size and mutation rate in *P. pacificus*, similar to *Caenorhabditis elegans*. In contrast to *C. elegans*, however, there is no evidence for a bias toward multistep mutations. The mutation spectrum of microsatellite loci in *P. pacificus* shows more insertions than deletions, indicating a tendency toward lengthening, a process that might have contributed to the increase in genome size. The mutation rates obtained for individual microsatellite markers provide guidelines for divergence time estimates that can be applied in *P. pacificus* next-generation sequencing approaches of wild isolates.

The nematode *Pristionchus pacificus* is a model organism increasingly used for integrative approaches in evolution biology, through interdisciplinary studies in evo-devo, population genetics, and ecology (Hong and Sommer 2006; Sommer 2009). *P. pacificus* has a generation time of 4 days in standard laboratory cultures (Sommer *et al.* 1996), well-developed tools for forward and reverse genetic analysis, DNA-mediated transformation (Schlager *et al.* 2009), and a fully sequenced genome (Dieterich *et al.* 2008). *Pristionchus* nematodes are unique among model organisms in their well-described necromenic association with scarab beetles, *e.g.*, *P. pacificus* has been found on *Exomala orientalis* in Japan and on *Oryctes borbonicus* on La Réunion Island in the Indian Ocean (Herrmann *et al.* 2007, 2010; Morgan *et al.* 2012). The *Pristionchus*−beetle association represents a robust platform for the isolation and characterization of new *Pristionchus* isolates on a global scale. Close to 30 *Pristionchus* species and more than 400 *P. pacificus* strains have been isolated between 2004 and 2011 in worldwide samplings, and a molecular phylogenetic framework has been generated (Mayer *et al.* 2007, 2009; Morgan *et al.* 2012).

Integrative approaches in evolutionary biology require a life history perspective. Specifically, robust evolutionary analyses depend on an understanding of the mutation patterns of different regions of the genomes (Lynch 2007). In a recent study, we used a mutation accumulation (MA) line approach to evaluate the pattern of mutations and to estimate the mutation rates of the mitochondrial genome of *P. pacificus* (Molnar *et al.* 2011). These can be used to resolve ancient evolutionary branches, whereas more recent evolutionary events are better studied using microsatellite regions. To most effectively use microsatellites for divergence estimation, we need to understand their mutation pattern and the factors that affect their mutation rate.

Microsatellites are DNA sequences composed of short units, no more than 6 bp long, found as tandem repeats throughout the genomes of most eukaryotic and prokaryotic organisms (Hancock 1999). They are ubiquitously but nonrandomly distributed in protein-coding and -noncoding regions (Toth *et al.* 2000). Their highly polymorphic nature made microsatellites the markers of choice in population genetics (Jarne and Lagoda 1996). Several mechanisms have been suggested to explain the high mutation rate of microsatellites, including errors during recombination, unequal crossing-over, and polymerase slippage during DNA replication (Schlötterer and Tautz 1992) or repair (Strand *et al.* 1993). Individual microsatellites are described as having a life cycle—they are born, they grow, and they die (Chambers and MacAvoy 2000).

Estimations of the rate and pattern of microsatellite mutations are usually indirect, based on allele frequency distributions (Chakraborty *et al.* 1997; Primmer and Ellegren 1998) or phylogenetic analyses (Jin *et al.* 1996; Dettman and Taylor 2004). The first studies aiming to understand the mutation mechanisms of microsatellites have been made possible by direct observations of mutations based on artificial constructs with expressed microsatellite sequences within bacterial and fungal systems (Levinson and Gutman 1987; Strand *et al.* 1993). Direct estimates of the microsatellite mutation rates are also derived from MA line experiments conducted in *Drosophila melanogaster* (Schug *et al.* 1997), *Caenorhabditis elegans*, *Daphnia pulex* (Seyfert *et al.* 2008), and *Arabidopsis thaliana* (Marriage *et al.* 2009). Under ideal conditions, MA line-based mutation rate estimates can be combined with genomic analysis of natural isolates of a given species and close relatives to provide robust divergence time estimates (Molnar *et al.* 2011).

Here, we evaluate the genomic composition of microsatellites for *P. pacificus* and make use of MA lines to provide robust estimates of the rate and size spectra of microsatellite mutations. We found that 0.59%–3.83% of the genome is composed of short tandem repeats. By analyzing 82 MA lines after 142 generations, we found a total of 31 mutations in these markers. There is a correlation between allele size and mutation rate, but no bias toward multistep mutations. We use these findings to suggest general guidelines for the selection of microsatellite markers in future genome-wide association studies of the evolutionary history of *P. pacificus*.

## Materials and Methods

### MA lines

The propagation of the MA lines has been described in detail previously (Molnar *et al.* 2011). To summarize, 100 MA lines were initiated from the F3 descendants of a single, inbred, wild-type *P. pacificus* PS312 laboratory strain. MA lines were propagated by a single, randomly chosen offspring from the middle of the reproduction period. Worms were cultured at 20° on nematode growth medium seeded with *Escherichia coli* as a food source, as originally described for *P. pacificus* (Sommer *et al.* 1996). Backup cultures were kept for two generations at 15° to prevent the accidental loss of lines. From the original 100 MA lines, 82 lines survived the 142 generations. Because a single homozygous progenitor started all the lines, a single random offspring began each generation and because *P. pacificus* is a self-fertilizing hermaphrodite, the variation between the strains is caused by the accumulation of mutations.

### Microsatellite pattern

Microsatellite loci were identified in the ‘Freeze 1’ assembly of the *P. pacificus* genome (available at www.pristionchus.org) using Tandem Repeats Finder (TRF) (Benson 1999) with two different sets of parameters: threshold alignment score 20 and alignment weights {2,7,7} ({match,mismatch,indels}) for the TRF-loose method and threshold alignment score 50 and alignment weights {2,3,5} for the TRF-strict method (Leclercq *et al.* 2007). The values for the weights can be 3, 5, and 7, with 3 being more permissive and 7 more restrictive. Leclercq *et al.* have previously shown that both the weights and threshold criteria influence the composition of the microsatellites detected (see also supporting information, Figure S1 for the differential screening results from the present study). Specifically, Leclercq *et al.* have shown that increasing TRF alignment score allows the detection of smaller and more perfect microsatellites, whereas decreasing the TRF weights allows for longer and more imperfect microsatellites to be detected. We, therefore, have created two data sets of microsatellite loci. Both datasets include perfect and imperfect repeats. The frequency of microsatellites was calculated as (number of microsatellite loci)/(number of nucleotides in megabases), considering the assembled genome of *P. pacificus* of 169 Mb, and loci of at least three repeat units. For a comparison base line, we screened the *C. elegans* genome with the same methods as for *P. pacificus*.

### Microsatellite markers for molecular analysis

We randomly chose 32 microsatellites and developed suitable primers (Table S1). These markers cover a range of three to 57 repeat units and have a percentage match score between 51 and 100. To ensure the evaluation of the mutation rate for the long microsatellites present in the genome, we chose nine additional markers for perfect tri-, tetra-, penta-, and hexanucleotide that had pattern repeat count greater than 30 (except the hexanucleotide locus that had the pattern repeat count of 17 but an overall length of more than 100 bp). We had 41 markers in total, represented by 19 perfect and 22 imperfect repeat loci.

### DNA extraction and amplification

For each MA line, genomic DNA was prepared from two full plates of worms, using worm lysis buffer (50 mM KCl; 10 mM Tris-HCl, pH 8.3; 2.5 mM MgCl_2_; 0.45% NP-40; 0.45% Tween-20; 5 µg/mL proteinase K). The suspension was incubated for 2 hr at 65°, followed by inactivation of the proteinase K at 95° for 10 min. All forward primers had an M13 tail (5′-CACGACGTTGTAAAACGAC-3′) attached at the 5′ end, labeled with 6-FAM, VIC, NED, or PET (Applied Biosystems) for genotyping (Table S1). We performed individual polymerase chain reactions in 20 µL of final volume for each marker, using an annealing temperature of 55°.

### Genotyping

Genotyping was performed on an ABI 3730*xl* using ABI Genemapper version 4.0 (Applied Biosystems) analysis software with the internal size standard GS500LIZ or GS1200LIZ. Markers M01 to M47 were multiplexed before genotyping. Markers M74 to M88 were labeled with 6-FAM and assessed individually. Mutations were detected with Genemapper version 4.0 (Applied Biosystems) by comparison to the progenitor of the MA lines and verified using independent DNA amplification and genotyping. All the assessed loci were homozygous.

### Mutation rate estimates

The mutation rates (per allele per generation) were calculated using the formula from Seyfert *et al.* (2008): µ = −[ln(1−*n*/*l*)]/*t*, where *n* is the number of mutations, *t* is the number of generations, and *l* is the number of lines. Note that the number of lines assessed may differ slightly between the markers.

## Results

### Tandem repeat pattern in *P. pacificus*

**Figure 1:**
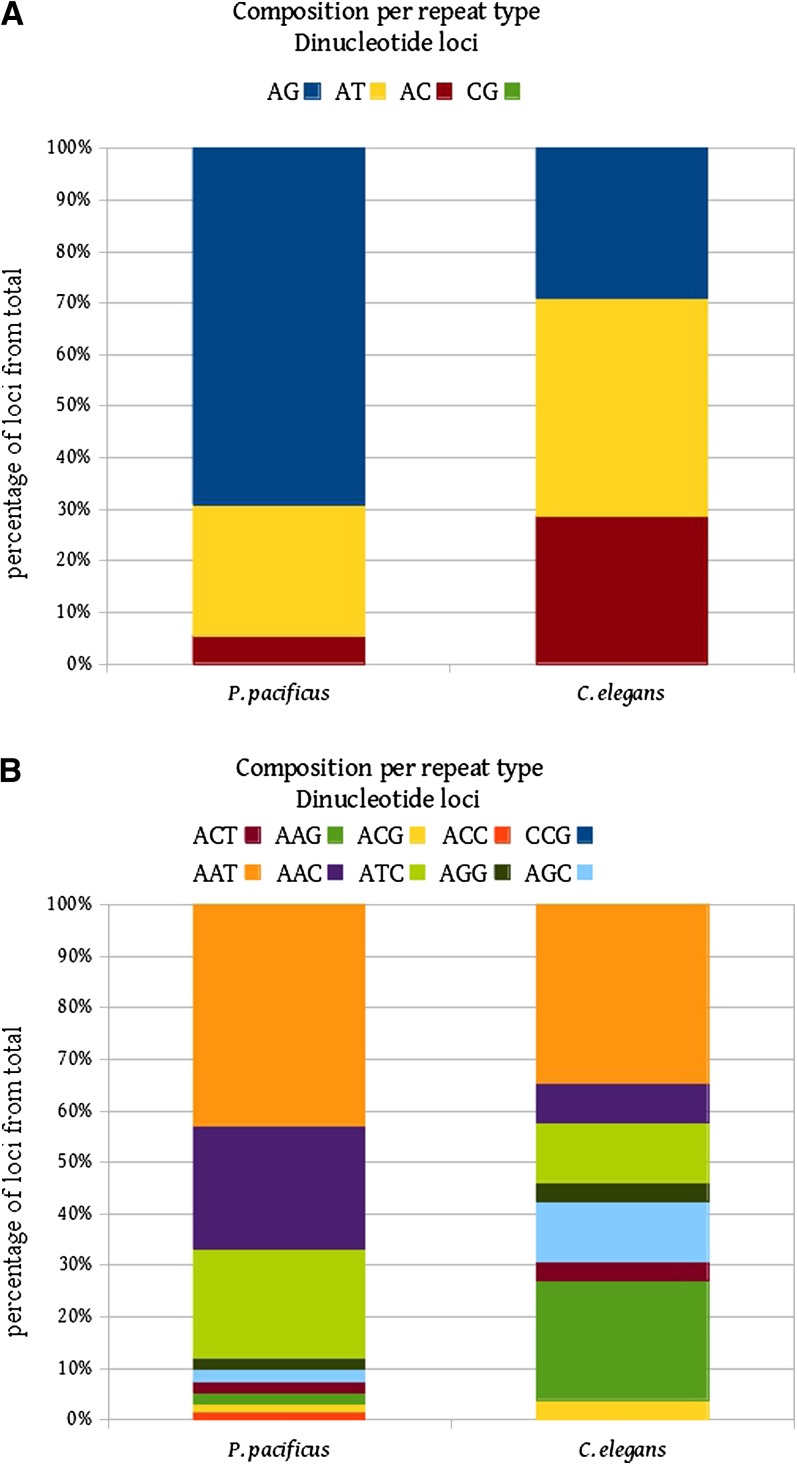
Composition per repeat type: (A) dinucleotide loci, (B) trinucleotide loci.

We screened the genome of *P. pacificus* for short tandem repeats consisting of di-, tri-, tetra-, penta-, and hexanucleotides with at least three repeat units, using the Tandem Repeats Finder software (Benson 1999). Data presented below always refer to duplex DNA, even if we show only the sequence of the repeated motif on one strand for simplicity, *i.e.*, notations like (AC)_n_ and (AC)_n_:(GT)_n_ are equivalent. The two sets of parameters, involving different thresholds and distinguishing between perfect and imperfect repeats (see *Materials and Methods*), yielded very different results. Specifically, in the genome of *P. pacificus*, the TRF-loose method counted 70,543 perfect loci, whereas the TRF-strict method identified only 730 loci. From the 730 perfect loci identified by the TRF-strict method, dinucleotide repeats are by far the most common repeat type (383/730). Of the four possible unique dimer combinations (AC, AG, AT, CG), three are present within the genome, with AG repeats representing the greatest number (265/383 loci; Table 1). In contrast, perfect CG repeats are not found in the *P. pacificus* genome. In comparison with the *C. elegans* genome, the dinucleotide repeats have a different repeat unit distribution, but the perfect CG repeats are also missing (Figure 1A).

**Table 1:** Observed repeat loci in the *P. pacificus* genome

	Perfect Repeats		Imperfect Repeats*^a^*	
Repeat unit	TRF–Strict	TRF–Loose	TRF–Strict	TRF–Loose
Total	730	Not all sites are mentioned		
Dinucleotides				
AC	21	1599	29	434
AG	265	10,785	1636	7510
AT	97	1541	316	647
CG	0	549	7	54
Total	383	14,474	1988	8645
Trinucleotides				
AAC	32	4155	632	1869
AAG	3	9336	606	5762
AAT	57	6437	629	3236
ACC	2	1093	8	1159
ACG	2	1858	21	399
ACT	3	1072	20	241
AGC	3	2762	359	1382
AGG	3	7247	573	4161
ATC	28	4461	79	1088
CCG	0	718	23	193
Total	133	39,139	2,950	19,490
Tetranucleotides				
AAAC	0	444	23	416
AAAG	1	902	38	1381
AAAT	4	2250	175	2921
AACC	0	4	0	36
AACG	0	174	0	70
AACT	2	86	7	63
AAGC	0	127	0	54
AAGG	0	1000	63	1616
AAGT	0	73	9	97
AATC	8	1457	63	1213
AATG	29	1426	122	1130
AATT	3	1222	835	2822
ACAG	0	239	70	205
ACAT	1	175	11	117
ACCC	0	68	218	26
ACCG	0	51	1	85
ACCT	0	82	3	57
ACGC	0	48	0	41
ACGG	0	46	1	35
ACGT	0	7	0	2
ACTC	1	309	3	223
ACTG	0	125	3	71
AGAT	2	325	8	240
AGCG	0	240	1	263
AGCT	0	8	0	0
AGGC	0	52	1	27
AGGG	1	995	0	2681
ATCC	0	330	0	283
ATCG	0	476	3	208
ATGC	0	44	0	19
CAGC	1	30	0	14
CCCG	0	38	0	54
CCGG	0	12	0	330
Total	53	12,922	1837	17,219
Pentanucleotides*^b^*				
AAAAG	2	80	16	878
AAAAT	1	312	86	1615
AAAGG	1	50	11	469
AAAGT	12	107	9	136
AAATC	4	101	11	236
AAGAG	1	316	201	2088
AAGGG	2	189	58	1100
AATAC	4	103	10	194
AATTC	55	334	48	453
ACATC	2	32	1	80
ACTCT	1	11	1	73
AGAGG	0	122	113	1274
AGGGG	0	139	167	1816
Total	85	Not all sites are mentioned		
Hexanucleotides*^b^*				
AAAAAC	1	56	66	338
AAAAAG	1	31	88	505
AAAAAT	0	46	70	790
AAAAGT	1	5	5	59
AAAATC	1	4	2	48
AAAGGT	2	158	5	226
AAATTC	11	18	5	59
AAATTG	20	47	11	95
AACAAT	2	36	203	457
AAGAGG	1	42	127	618
AAGCCT	8	43	68	101
AAGTAT	3	21	12	27
AATAAG	6	24	101	207
AATATC	0	32	2	33
AATCTG	1	3	1	17
AATTAC	6	27	9	55
ACACGC	1	1	2	12
ACCAGG	1	1	0	2
ACTCGC	1	1	210	892
AGAGGG	0	78	6	14
AGCCGG	1	2	0	9
AGAGTC	1	1	10	45
ATCTGT	2	50	7	45
ATCGTC	2	5	6	34
ATCTTC	1	14	83	212
ATGATT	2	4	9	57
Total	76	Not all sites are mentioned		

a Shown only if perfect loci were found.

b Shown only if perfect loci were found with the TRF-strict method or more than 30 perfect loci were found with TRF-loose method.

A total of 133 loci were found representing the 10 possible unique trimer combinations. Trimers rich in AT are the most common, with [(A/T)_2_, X_1_] combinations accounting for 92% of the instances found (Table 1). Again, one possible combination is not found, and this is the pure CCG repeat (Table 1, Figure 1B). There are 33 unique tetramer combinations possible, 11 of which have been found in the *P. pacificus* genome. 90% of the tetramer repeats are represented by [(A/T)_3_,X_1_] type of repeats. Most of the repeat units with more than 50% CG-content ([(A/T)_1_(C/G)_3_] and [(A/T)_2_(C/G)_2_]) were missing, including [(C/G)_4_]. Similarly, 90% of the pentamers and 72% of the hexamers are represented by AT-rich motifs ([(A/T)_4_,X_1_ and [(A/T)_5_,X_1_], respectively; Table 1). The ‘nematode’ telomeric repeat motif, (TTAGGC)_n_ (Niedermaier and Moritz 2000), is found to be the most abundant among the hexamer repeats (8 pure loci in TRF-strict dataset—the most in terms of number of loci per repeat type and the longest repeats per locus), but no functional conclusion can be drawn.

The TRF-loose method yielded in general the same trend for the different types of repeats, with AT-rich loci representing more than 50% of the total number of loci found for each repeat unit size. CG-rich pentamer and hexamer repeats are few or missing (Table 1).

### Mutation rates

We randomly selected 32 markers from the loci found with the TRF-loose method (the larger dataset). To these we added nine markers selected based on the total repeat count of more than 30 to have the longest loci represented in the analysis. Among the 41 loci assayed in the 82 MA lines, we found 31 mutation events at 11 loci (Table 2). At four of these 11 loci, only a single mutation was observed, which is insufficient for deriving proper mutation frequencies. Therefore, most conclusions listed below are based on markers with more than one mutation. Several general trends can be observed in the mutation patterns. First, M17 was the only locus smaller than 17 units that contained a mutation. In general, microsatellite loci that contain more than 30 repeat units show more mutations than the shorter ones, an observation similar to the *C. elegans* MA line-based analysis of microsatellites mutation rates (Seyfert *et al.* 2008). Second, 26 of the 31 mutations involved gain or loss of a single repeat unit, with insertions outnumbering deletions (21 insertions and 5 deletions). Only five of the 31 mutations involved a change of more than one repeat unit, and only one large deletion (−9 repeats at the M78) occurred at a locus with more than 30 repeat units. Third, most of the mutations were found in perfect, long microsatellite loci (29 of 31 mutations; Table 2). Fourth, in the overall MA pattern, deletions are unable to balance out the insertions (8 *vs.* 23 repeat units). This observation suggests that the microsatellite loci have a tendency toward lengthening. Further analysis shows that seven of the analyzed loci (M34, M77, M79, M80, M82, M83, and M84) show accumulated growth, two (M17, M78) show accumulated decrease in length, and two (M74, M88) show no accumulated change in size. Finally, the mutation rate per locus per generation in *P. pacificus* ranges from 8.9 × 10^−5^ to 7.5 × 10^−4^ for those markers where mutations occurred.

**Table 2:** Allelic mutation rate estimates per generation

Marker	Chr.	Repeat	Percent Match	No. Mutations	Magnitude of Mutation (No. Lines)	Mutation Rate*^a^*
M21	II	(GGGCAC)_11_	51	0		
M01	I	(CT)_55_	55	0		
M15	II	(TC)_57_	55	0		
M41	V	(TCT)_26_	59	0		
M07	I	(TTG)_18_	64	0		
M43	V	(TTAA)_15_	69	0		
M29	III	(TTAA)_10_	72	0		
M16	II	(TGA)_15_	73	0		
M35	IV	(CTCC)_20_	73	0		
M42	V	(AATT)_9_	75	0		
M13	I	(CTTAAC)_6_	78	0		
M08	I	(TAAT)_3_	81	0		
M47	X	(GT)_7_	84	0		
M06	I	(AAC)_16_	85	0		
M34	IV	(AT)_17_	85	1	+1(1)	(9.2 × 10^−5^)
M26	III	(TTA)_29_	85	0		
M04	I	(CAA)_11_	87	0		
M22	II	(GAATAA)_5_	88	0		
M45	V	(GAGAG)_3_	91	0		
M11	I	(TTCTT)_3_	92	0		
M33	IV	(AG)_17_	93	0		
M14	I	(GAATAA)_4_	95	0		
M28	III	(TGG)_3_	100	0		
M18	II	(TCT)_3_	100	0		
M17	II	(GTT)_3_	100	1	−1(1)	(8.9 × 10^−5^)
M19	II	(TCGA)_3_	100	0		
M38	IV	(AAGCCT)_6_	100	0		
M03	I	(TC)_7_	100	0		
M05	I	(AGT)_8_	100	0		
M02	I	(GA)_13_	100	0		
M25	III	(AG)_14_	100	0		
M46	X	(AG)_15_	100	0		
M84	III	(CTCTTC)_17_	100	1	+1(1)	(8.9 × 10^−5^)
M74	V	(CAA)_33_	100	2	−1(1), +1(1)	2.3 × 10^−4^
M77	X	(ACAT)_35_	100	1	+1(1)	(9.6 × 10^−5^)
M78	I/V	(ACAT)_36_	100	3	+1(2), -9(1)	2.8 × 10^−4^
M79	I	(TTAG)_40_	100	4	+1(4)	3.6 × 10^−4^
M82	X	(TGAAT)_43_	100	6	−3(1), -1(1), +1(2), +2(1), +3(1)	7.5 × 10^−4^
M88	?	(TC)_59_	100	3	−1(2), -2(1)	2.7 × 10^−4^
M80	?	(CAGC)_64_	100	2	+1(2)	2.1 × 10^−4^
M83	?	(TTCAA)_64_	100	7	+1(7)	6.5 × 10^−4^

a Mutation rate is calculated per locus per generation (the number of lines assessed for each marker may differ). Mutation rates given in parentheses are based on single mutation events.

## Discussion

This is the first analysis of mutational processes and mutation rate estimates in the nuclear genome of *P. pacificus* because previous knowledge is based solely on the mitochondrial genome (Molnar *et al.* 2011). We have analyzed the tandem repeat pattern of the *P. pacificus* genome and studied the spontaneous mutation rates for microsatellite markers. From the mutation patterns and mutation rates obtained for individual microsatellite markers, we provide guidelines for the properties of microsatellite markers to be useful for divergence time estimates in future genome-wide sequencing projects (to follow in this section).

*P. pacificus* and *C. elegans* belong to the same nematode clade but they are only distantly related, representing members of different nematode families (Dieterich *et al.* 2008). Sequence turnover over these evolutionary distances resulted in unrelated microsatellite patterns in these two species. Therefore, the microsatellites are not homologous and cannot be directly compared, which unfortunately prevents the usage of statistical methods in a meaningful manner. The average overall AT content for the *P. pacificus* genome is 58% (Dieterich *et al.* 2008); therefore, we would expect AT dinucleotide repeats to be more common, followed by AC and AG in approximate equal numbers, and fewer CG loci. This pattern is, however, not followed by the dimer repeats composition found in *P. pacificus* genome, arguing against the expectation of cryptic simplicity. In contrast, the tri- to hexanucleotide repeat loci do follow this expectation, with AT-rich repeats being more abundant than the others. The most striking finding, however, is the absence or near absence of the CG loci. Direct and indirect observations tend to support the stepwise mutation model at microsatellite loci (Schlötterer and Tautz 1992; Weber and Wong 1993; Thuillet *et al.* 2002), by which their sequence is altered by addition or deletion of one repeat at a time. An alternative is the model according to which the sequence of microsatellites can be altered by large deletions, due to secondary structures that certain types of repeats can form (Di Rienzo *et al.* 1994). The absence of perfect CG dimer repeats but the presence of impure and CG-rich loci might support the latter model of microsatellite evolution.

**Figure 2:**
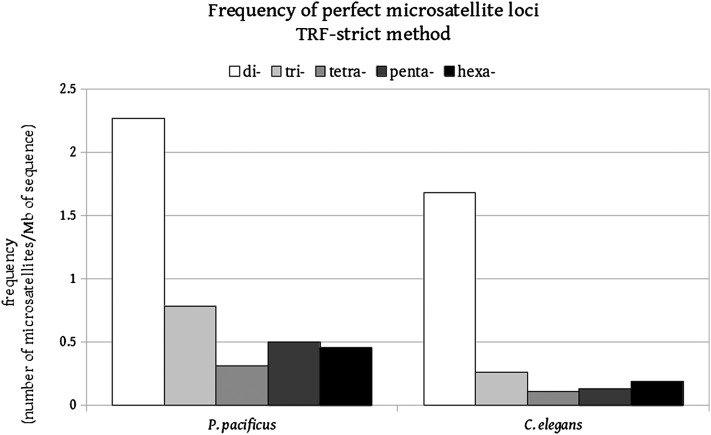
Frequency of perfect microsatellite loci, TRF-strict method.

A comparison with the *C. elegans* microsatellite dataset reveals that *P. pacificus* has an overall greater frequency of perfect microsatellite loci, although the dinucleotide repeats dominate the landscape in both genomes (Figure 2), a finding that has also been made by Castagnone-Sereno *et al.* (2010) using different algorithms. In a second step, we evaluated the mutation rate at microsatellite loci ranging from di- to hexanucleotide repeats, randomly chosen in the noncoding genome of *P. pacificus*. The random choice allows us to avoid a bias by assaying only certain types of repeats. However, it does not allow us to make decisions of how the mutation rate is influenced by the repeat unit size, nucleotide composition, or the overall length of the locus. Although the loci have been chosen randomly, eight of 10 dinucleotide loci are of the type (AG)_n_. This correlates with the general composition of the genome of *P. pacificus*, which has more AG repeats than other dinucleotide repeats.

The mutation patterns observed for *P. pacificus* in this study support the idea that mutational processes are length dependent. Specifically, large loci have, on average, more mutations than the small ones (three mutations in a (X)_3_ locus *vs.* seven mutations in a (X)_64_ locus). A second general trend, supported by the *P. pacificus* data, is that imperfect loci are less prone to accumulate mutations than the perfect ones. From 22 loci with diverse percentage of match and with a wide range of repeat unit size tested, only one showed a mutation (M34; Table 2). A comparison of the mutation rates at microsatellite loci with the same range of repeat number between *C. elegans* (Seyfert *et al.* 2008) and *P. pacificus* shows a similar effect of repeat number on mutation rates in both organisms.

A third major trend in the *P. pacificus* dataset is that the mutation process is upwardly biased in that loci tend to gain units more frequently that they lose units. Similar trends have been shown previously in other organisms (Primmer *et al.* 1996; Ellegren 2000). At the same time, long alleles tend to contract upon mutation (Harr and Schlötterer 2000; Xu *et al.* 2000). The mutations observed in *P. pacificus* are 21 insertions and 5 deletions, showing that microsatellites in *P. pacificus* have a tendency toward lengthening. It is interesting to note that the largest *P. pacificus* microsatellites detected in the genomes assembly are substantially smaller than the largest *C. elegans* microsatellites, which have repeat units greater than 68 (Seyfert *et al.* 2008). In *P. pacificus*, the loci M80 and M83, both show insertions, indicating that they are still in the growth phase. We speculate that the *P. pacificus* microsatellites, on average, are still in the expansion phase, a process that might have substantially contributed to the increase of the *P. pacificus* genome size relative to *C. elegans*. A final aspect of our analysis is that the *P. pacificus* genome shows no evidence for a bias toward multistep mutations. Specifically, all but five mutations are single-step insertions or deletions. This pattern is clearly distinct from what has been observed in *C. elegans*, indicating, again, the species and locus-specificity of the mutational processes.

The rate and pattern of mutations observed in the MA lines have implications for the use of microsatellites for inference of genetic history. It is critical to recognize that the evolutionary rate for a single locus will change with the size of the allele. Thus, choosing a microsatellite locus with the appropriate evolutionary rate to address a specific evolutionary time frame requires a careful consideration of allele size ranges. The data presented in this study provide guidelines for the selection of adequate markers for studying recent and ancient evolutionary branches of *P. pacificus*. Specifically, the absence of mutations in many short and/or imperfect loci, as well as the fact that for five markers only one mutation has been identified, do not allow us to use these results in deriving a mean mutation frequency. Interestingly however, all but one of the perfect repeats with more than 30 repeat units recovered multiple mutation events that resulted in a quite stable mutation rate of 2.5−7.5 × 10^−4^ (Table 2). Therefore, we suggest to only use microsatellite markers with a minimal length of 30 repeat units in studies that aim to reconstruct the evolutionary history of wild isolates. Furthermore, we suggest that such studies should use an average mutation frequency of 5 × 10^−4^ given the relatively stable mutation frequencies obtained in this study. Taken together therefore, this study provides useful information for future genome-wide studies that investigate the evolutionary history of *P. pacificus*.

## Supplementary Material

Supporting Information
