# Supplementary material for: Tandem-Repeat Patterns and Mutation Rates in Microsatellites of the Nematode Model Organism Pristionchus pacificus
Source: G3 (Bethesda). 2012 Sep 1;2(9):1027–34. doi: 10.1534/g3.112.003129 (PMC3429916; doi:10.1534/g3.112.003129)
Supplement: Supporting Information [file supp_2_9_1027__index.html]

Supporting Information 

# Tandem-Repeat Patterns and Mutation Rates in Microsatellites of the Nematode Model Organism *Pristionchus pacificus*

## Supporting Information for Molnar *et al.*, 2012

**Files in this Data Supplement:**

- Supporting Information - Figure S1 and Table S1 (PDF, 193 KB)
- Figure S1 - Differential screening results (PDF, 173 KB)
- Table S1 - Microsatellite loci assayed in *P. pacificus* (PDF, 69 KB)
