# Supplementary material for: Tandem-Repeat Patterns and Mutation Rates in Microsatellites of the Nematode Model Organism Pristionchus pacificus
Source: G3 (Bethesda). 2012 Sep 1;2(9):1027–34. doi: 10.1534/g3.112.003129 (PMC3429916; doi:10.1534/g3.112.003129)
Supplement: Supporting Information [file supp_2.9.1027_003129SI.pdf]

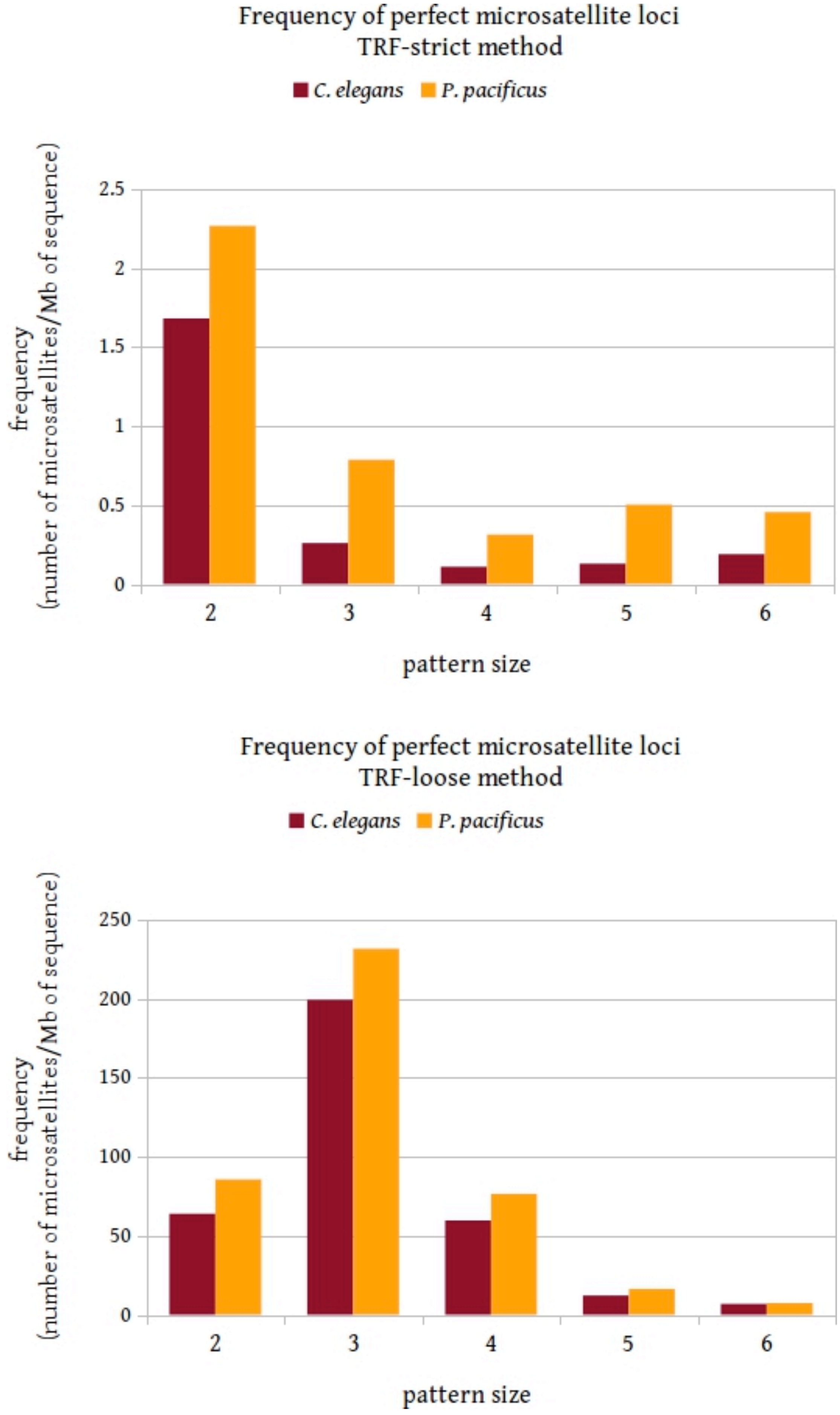

Figure S1 Differential screening results.

Table S1 Microsatellite loci assayed in *P. pacificus*

Marker (Dye)	Chr	Repeat	Percent match	Forward primer 5'- 3'/ Reverse primer 5'- 3'
			Randomly chosen markers	
M01 (6-FAM)	I	$(CT)_{55}$	55	AAAGCAATGCCAGAAGGAAA / TTCTTGCCGTACAAAGAATGA
M02 (NED)	I	$(GA)_{13}$	100	TTGGCTTTCTACAGCTAAATCG / GCCTGTGAAGGGGATATTGA
M03 (VIC)	I	$(TC)_{7}$	100	GTTTGTCAGCCCGATATGGT / GGGATGGGAAGGGAGAAAC
M04 (VIC)	I	$(CAA)_{11}$	87	GCTGGCATTGGAAAGAAAGT / AAGCCGATCAATCTCTGTCA
M05 (PET)	I	$(AGT)_{8}$	100	CGATTCCGTCGCTGTTTTC / AAGCCGATCAATCTCTGTCA
M06 (PET)	I	$(AAC)_{16}$	85	AGGCTCGTGTCGTTCATGTT / GCCAGCTACGGATGATTGAT
M07 (PET)	I	$(TTG)_{18}$	64	GCACAATCGGGTCTGAAAAT / TCCGGAACCTACAAAAGTGG
M08 (PET)	I	$(TAAT)_{3}$	81	GGTAAGTGCGCTGGAGAGAG / GCCCGAGGACACCTACTACA
M11 (6-FAM)	I	$(TTCTT)_{3}$	92	AACCGAATGTGGACGAAGAG / GGTCATCCCTTGTTTGTGCT
M13 (VIC)	I	$(CTTAAC)_{6}$	78	AAAGGCGAGGGTACAGTCCT / CGAGCAACTGAAATGAACGA
M14 (PET)	I	$(GAATAA)_{4}$	95	CTCCCTACCTCCCACATCAA / ATACTCGGTGGCCAGTATGC
M15 (VIC)	II	$(TC)_{57}$	55	CGGCTTCCTTCCTCTCTTCT / CCAACTCTCCCCTTTTCACA
M16 (PET)	II	$(TGA)_{15}$	73	GCCTGGACAAAATTGTCGTT / CGTTGTGGTGTGAGTTACGG
M17 (VIC)	II	$(GTT)_{3}$	100	AAGACCTGGGCATCAAACTG / ACGACCGAAACATCTTGGAC
M18 (VIC)	II	$(TCT)_{3}$	100	TCTAACTGGATGCCGGAACT / AGCGGTGTTAATGAGCGTTT
M19 (NED)	II	$(TCGA)_{3}$	100	ACATTGTCCATTCAGCTTCG / CCGAAAGAGAGACCATCTCC
M21 (6-FAM)	II	$(GGGCAC)_{11}$	51	GGAAAGGAGAGGGGAGTCTG / TCAGCCTCCTCAGGTAATGA
M22 (6-FAM)	II	$(GAATAA)_{5}$	88	ATTACGAGCTGGCCAATCAG / AAGTGCTACACTCGGTGCAA
M25 (NED)	III	$(AG)_{14}$	100	AGCAACGGCTCAATCAAAGT / GCAGTGACTCTTTCCGGTTT
M26 (VIC)	III	$(TTA)_{29}$	85	CTGATTGCCCGACAACATTC / CCAACGCATCACTATGGCTA
M28 (6-FAM)	III	$(TGG)_{3}$	100	GATTCGGTGGTGTAGTCGATG / TTCTGTGCATGACGAAGTTTCT
M29 (6-FAM)	III	$(TTAA)_{10}$	72	GTGTCGTGTGCCGACTAAGA / CTCTCCGGTTCCTCCTCTCT
M33 (6-FAM)	IV	$(AG)_{17}$	93	TGATTGACTGAGGGCTTTCC / ATGCCACCCTTCTGATTGAC
M34 (6-FAM)	IV	$(AT)_{17}$	85	ATTTGTAGGGGAAGGGGTTG / CATAAACGGTGACAGGCACA
M35 (6-FAM)	IV	$(CTCC)_{20}$	73	TTGGTCTTCTCCCCTTCTGA / TCTCCTTGTTTCCCCTCCTT
M38 (6-FAM)	IV	$(AAGCCT)_{6}$	100	CTCAATCAGGGAGGAACCAA / CCTCTCCCTCTCGACAACTG
M41 (NED)	V	$(TCT)_{26}$	59	CATCAATGAAACCCCATTCC / ATAACAGGCGCTGCTCTCTC
M42 (6-FAM)	V	$(AATT)_{9}$	75	GAAACACGAATGCCCACTCT / TCAGAGATGCCCGAGAGTTT
M43 (6-FAM)	V	$(TTAA)_{15}$	69	CTCCATCGCCTTCTTCTTTG / CCGGTCTATTCCTGGTCGTA
M45 (PET)	V	$(GAGAG)_{3}$	91	ATAGAAGAAGCGGTGGGTCA / CTCCACGTTCACTCGTCTCA
M46 (PET)	X	$(AG)_{15}$	100	CGCAATGAAGAGAACGAGGT / GAGCCGAGAAGTCCAGTGAG
M47 (6-FAM)	X	$(GT)_{7}$	84	GTCGGTCAAATATCGCCTGT / CGTCTGTCCCCGTTATCACT
			Tri- to hexanucleotide microsatellites with more than 30 repeat count	
M74 (6-FAM)	V	$(CAA)_{33}$	100	CCTCAGTCCCAACAGCAGAT / TTCCCAACACTTTGCATGAG
M77 (6-FAM)	X	$(ACAT)_{35}$	100	GGATCGTCCCTTTCGTCATA / CACGAAACACACAGCGTTCT
M78 (6-FAM)	I/V	$(ACAT)_{36}$	100	GGTCTGAGGGGTGTACGATG / TCATCATTGCCGAATTGTGT
M79 (6-FAM)	I	$(TTAG)_{40}$	100	AAAGTCGAGCGTGACAGACA / AATACGCATCTCGCTCTGGT
M80 (6-FAM)	?	$(CAGC)_{64}$	100	AAAGGGGTGAAATTTGCATT / GAGAAGTATGCAGGGCTGTTG

M82 (6-FAM)	X	$(TGAAT)_{43}$	100	TTGCAGGTTCGATACATTCTTC / GTTTCCCCCTGCAGGTCTAT
M83 (6-FAM)	?	$(TTCAA)_{64}$	100	GAAAATGTCGCCACGAAAAT / TGCAAACGTTACACAAACAGC
M84 (6-FAM)	III	$(CTCTTC)_{17}$	100	ACGATGATGATGTGCGTGAG / AATCGTCCCTTCCCCTGTAG
