# Supplementary figures and images for: Tandem-Repeat Patterns and Mutation Rates in Microsatellites of the Nematode Model Organism Pristionchus pacificus
Source: G3 (Bethesda). 2012 Sep 1;2(9):1027–34. doi: 10.1534/g3.112.003129 (PMC3429916; doi:10.1534/g3.112.003129)

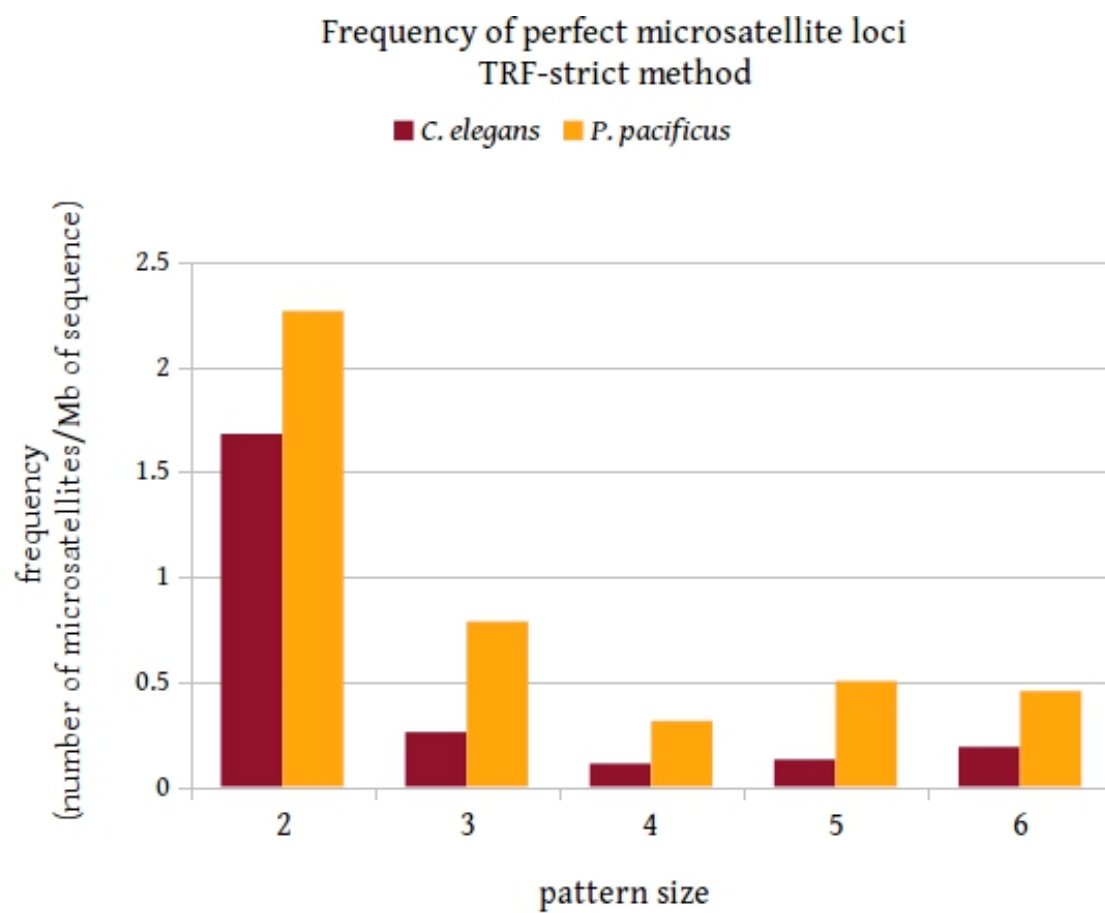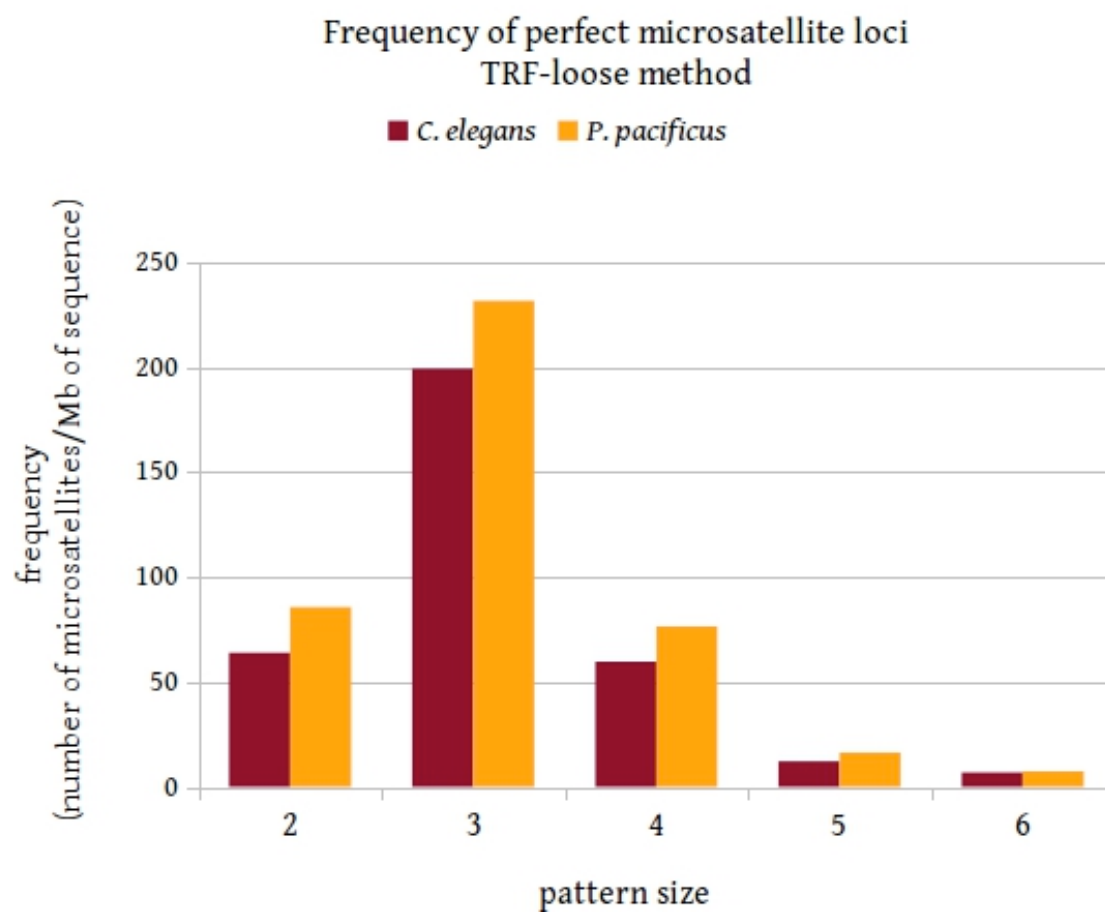

**Figure S1** Differential screening results.

Supplement: Supporting Information [file supp_2.9.1027_FigureS1.pdf]
